# Global Positioning from a Single Image of a Rectangle in Conical Perspective

**DOI:** 10.3390/s19245432

**Published:** 2019-12-10

**Authors:** Manuel Estrems Amestoy, Óscar de Francisco Ortiz

**Affiliations:** 1 Mechanics, Materials and Manufacturing Engineering department, Technical University of Cartagena, 30202 Cartagena, Spain; manuel.estrems@upct.es; 2 Department of Engineering and Applied Technologies, University Center of Defense, San Javier Air Force Base, MDE-UPCT, 30720 Santiago de la Ribera, Spain

**Keywords:** conical perspective, dihedral projection, positioning, trigonometry

## Abstract

This article presents a method to obtain the overall positioning of the focus of a camera from an image that includes a rectangle in a fixed reference with known position and dimension. This technique uses basic principles of descriptive geometry introduced in engineering courses. The document will first show how to obtain the dihedral projections of a rectangle after three turns and one translation. Secondly, we will proceed to obtain the image of the rectangle rotated in a conical perspective, taking the elevation plane as the drawing plane and a specific point in space as the view point, and represented in the dihedral system. Thirdly, we proceed with the inverse perspective transformation; we will expose a method to obtain the coordinates in the space of a rectangle obtained from an image. Finally, we check the method experimentally by taking an image of the rectangle with a camera in which the coordinates in the drawing plane (center of the image) are the only available position information. Then, the positioning and orientation of the camera in 3D will be obtained.

## 1. Introduction

Pose determination is to estimate the position and orientation of one calibrated camera using a set of correspondences between 3D control points and 2D image points [1]. Determination of surface orientation has important applications such robotics, object recognition, 3D measurement or tracking of moving objects. Magee [2] was the first to present a procedure for determining the unique position of a robot in a three dimensional space. That method has been continuously improved in different areas as large non-cooperative satellites [3] or Unmanned Aerial Vehicle (UAV) Control [4,5]. Different methods for monocular pose estimation have been studied in the past [6,7,8,9]. More recently, marker-based positioning systems as ArUco, Chilitags, ApriTags, or ArToolKit, among others, have been introduced to estimate quantitative changes in distances and orientations in many technological applications, such as autonomous robots [10,11,12], unmanned vehicles [13,14,15,16], or virtual assistants [17,18,19,20].

The calibration and orientation of a camera from its images has been obtained through different approaches in the past with good precision through techniques such as using a single image with four coplanar control lines [21], three coplanar circles [22], using parallelogrammatic grid points [23], or even using only three points in the world coordinate system when a multiple camera system is used [24]. Becker [25] introduced a new technique using an iterative method which solves the parameters that minimize vanishing point dispersion to solve for radial and decentering lens distortion directly from the results of vanishing point estimation, precluding the need for special calibration templates. Single image based reconstruction has been deeply studied by many authors such as Delage [26], Wilczkowiak et al. [27], Sturm and Maybank [28] or Micusik et al. [29], assuming perpendicularity and parallelism to recover the lack of information. Other authors such as Penna [30] showed that there is sufficient information in the two-dimensional perspective projection of an arbitrary quadrilateral of known shape and size in three-space to determine the exact three-dimensional coordinates of its vertices, generalizing known results for rectangles. Duan [1] used the projection of a trapezium for pose estimation and plane measurement in a very simple way. An iterative algorithm was used by Hong & Yang [31] to establish the relationship between parameters and the world coordinates of a given 3D calibration point. Nevertheless, additional studies via rectangular structures as in Haralick [8] or Wefelscheid [32] use similar concepts with a different approach. In contrast, our research used the information provided by the dihedral projections of a rectangle to determine the image of the rectangle rotated in a conical perspective.

Computer vision has been used in areas, such as unmanned vehicles, to estimate relative 3D position and altitude using algorithms based on four feature points, such as square and parallel relations, to avoid complicated calculations [33]. An algorithm for pose estimation based on volume measurement of tetrahedra composed of target points and the lens center of the vision system was proposed by Abidi [6]. 3D model reconstruction from a single image calibrating a camera and recovering the geometry and the photometry of objects was part of Guillou’s [34] research and a novel method to find the initial solutions for iterative camera pose estimation using coplanar points was provided by Zhou [35]. A general photogrammetric method for determining object position and orientation was presented by Yuan [36]. Recently Wang et al. [37] studied active relocalization of a 3D camera pose from a single reference image; a recent and challenging problem in computer vision and robotics. Pose estimation of smooth metal parts is an important task in intelligent manufacturing. Ulrich [38], Sakcak [39], Han [40] and He [41] proposed a solution using a monocular camera and corresponding practical algorithms.

The adjustment of tools in machining centers is usually the slowest and most critical operation in the positioning of the machined parts. The provision of a tool that includes machine displacements and images with edge detection can be adjusted at micrometric scales without the need for lasers or probes. Other possible application could be the metrology by vision, since in the case of characteristics to be measured in the same plane of a rectangle of known dimensions, the dihedral perspective of the aforementioned characteristic can be obtained and non-contact metrological checks can be performed immediately (in real time) compensating many of the existing errors. This is an essential aspect to achieve the efficiency and flexibility required by controls in production systems in Industry 4.0.

In this work a new method to obtain the camera coordinates of a rectangle from its image is proposed. This method is based in the principles of descriptive geometry as developed by Monge [42], which is studied in basic engineering courses. In order to explain the method a remembrance of the construction of a rectangle in conical perspective is described, and an inverse path is proposed. Finally, an experiment has been designed to check the precision of the method.

## 2. Dihedral Projection of a Rectangle. Rotations and Translations

In this case the problem input data is the dihedral projection of a rectangle in which the length of one side L is known. Therefore, it is represented by its coordinates $x^{*}$ and $z^{*}$. This rectangle is rotated by three angles ϕ, ξ, and θ. The transformation matrices are applied to obtain a global rotation matrix and the translation is made to the point $X_{0}$, the coordinates of the vertices are then obtained and presented in a table of dihedral information. The Top View of the dihedral would be represented by the xy plane, and the elevation of the dihedral is the xz plane. The projections of the rectangle on both planes will be its dihedral representation [42,43].

## 3. Conical Projection

**Figure 1:**
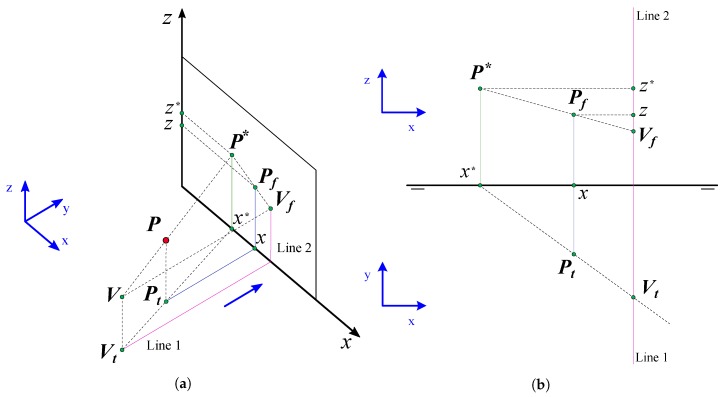
Plotting a point (P) in a conical perspective from its dihedral projection. Subindex *f* refers to the Front View and subindex t refers to the Top View. (**a**) Point dihedral construction view; (**b**) Dihedral projections.

With the point of view with coordinates $\left(V_{x},V_{y},0\right)$ and represented in the same dihedral system as the rectangle, where the Front View coincide with the image plane and $V_{y}$ is the focal distance, the vertices coordinates $\left(x^{*},z^{*}\right)$ in the rectangle in the conical perspective are obtained. The method used consists in creating, from the Top View, a line that passes through $\left(V_{x},V_{y},0\right)$ and the Top Projection of the point *P*$\left(P_{x},P_{y},0\right)$ obtaining the intersection with the image plane which will be the coordinate $x_{p}^{*}$. This coordinate $x_{p}^{*}$ is calculated by drawing the line that passes through $\left(V_{x},V_{y},0\right)$ and the Front View of the point *P*$\left(P_{x},P_{y},0\right)$ and obtaining the intersection with the vertical line that starts at $x^{*}$. Consequently, the conical projection of the point in the image plane with coordinates $\left(x^{*},z^{*}\right)$ is calculated. When this operation is performed Figure 1 with the four points in the rectangle, the rectangle in conical perspective is obtained.

## 4. Obtaining the Possible Front View and Top View of Dihedral Projection of the Rectangle

**Figure 2:**
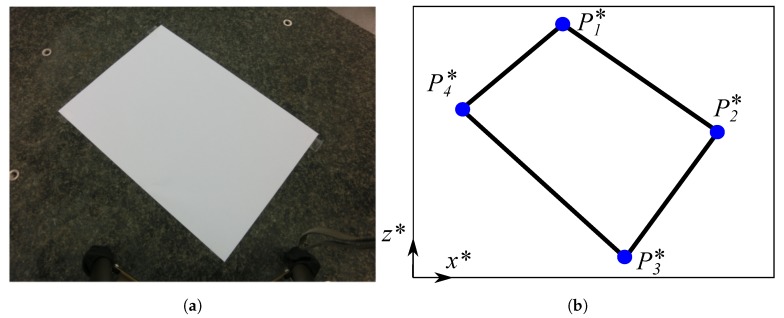
Actual conical perspective of the object froma photograph: (**a**) Initial image; (**b**) Referencemodel.

Using the coordinates in the conical perspective and knowing the projection of the point of view in the Front and Top planes, the coordinates of the edges of the rectangle are calculated (Figure 2).

To do this, we use part of the geometric method described by Wefelscheid et al. [32] obtaining auxiliary points that help us calculate the dihedral projection from the conical projection. These auxiliary points are:

1. Point $M^{*}$. Intersection between the lines joining $P_{1}^{*}P_{3}^{*}$ and $P_{2}^{*}P_{4}^{*}$.
2. Vanishing point $V_{1}^{*}$ as intersection between the lines $P_{1}^{*}P_{2}^{*}$ and $P_{3}^{*}P_{4}^{*}$.
3. Vanishing point $V_{2}^{*}$ as intersection between the lines $P_{1}^{*}P_{4}^{*}$ and $P_{2}^{*}P_{3}^{*}$.
4. Midpoint of edges $P_{12}^{*}$, $P_{23}^{*}$, $P_{34}^{*}$, $P_{14}^{*}$ as intersection of the lines that are drawn from the vanishing point to $M^{*}$ with the respective edges.

**Figure 3:**
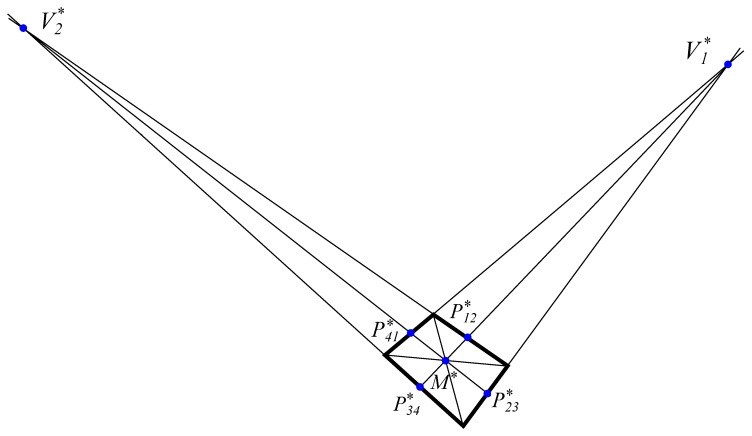
Obtaining auxiliary points from the four vertices of the rectangle.

The auxiliary points are represented in Figure 3.

These operations can be done graphically by drawing on paper, so it is computationally reduced to intersections between lines that are defined each by two points as in Table 1 as represented in Figure 3.

After obtaining these points, a proposal of the Front View of the rectangle is based on two graphic properties:

1. The points of the Front View projection are in the lines that start from the center point *V* whose coordinates are ($V_{x},0,V_{z}$) and go though the point of the image $P_{1}^{*}$, $P_{2}^{*}$, $P_{3}^{*}$, $P_{4}^{*}$, $M^{*}$, $P_{12}^{*}$, $P_{23}^{*}$, $P_{34}^{*}$, $P_{14}^{*}$.
2. In the dihedral projection the center points are in the geometric center of the segment of the side, dividing this side in two. For example, $P_{12}$ is in the center point of the segment that joins $P_{1}$ and $P_{2}$.
3. Opposite sides are parallel in the dihedral projection.

**Figure 4:**
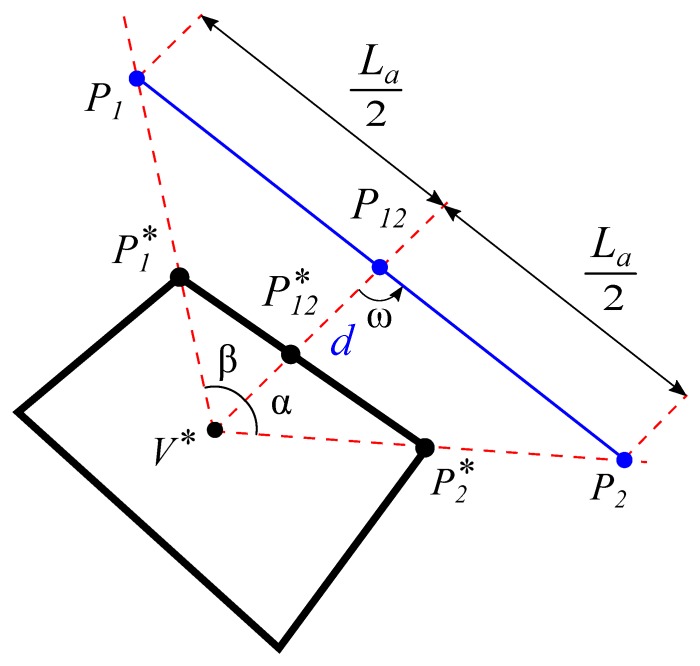
Line orientation that subdivides the segment of the side in two equal segments.

**Figure 5:**
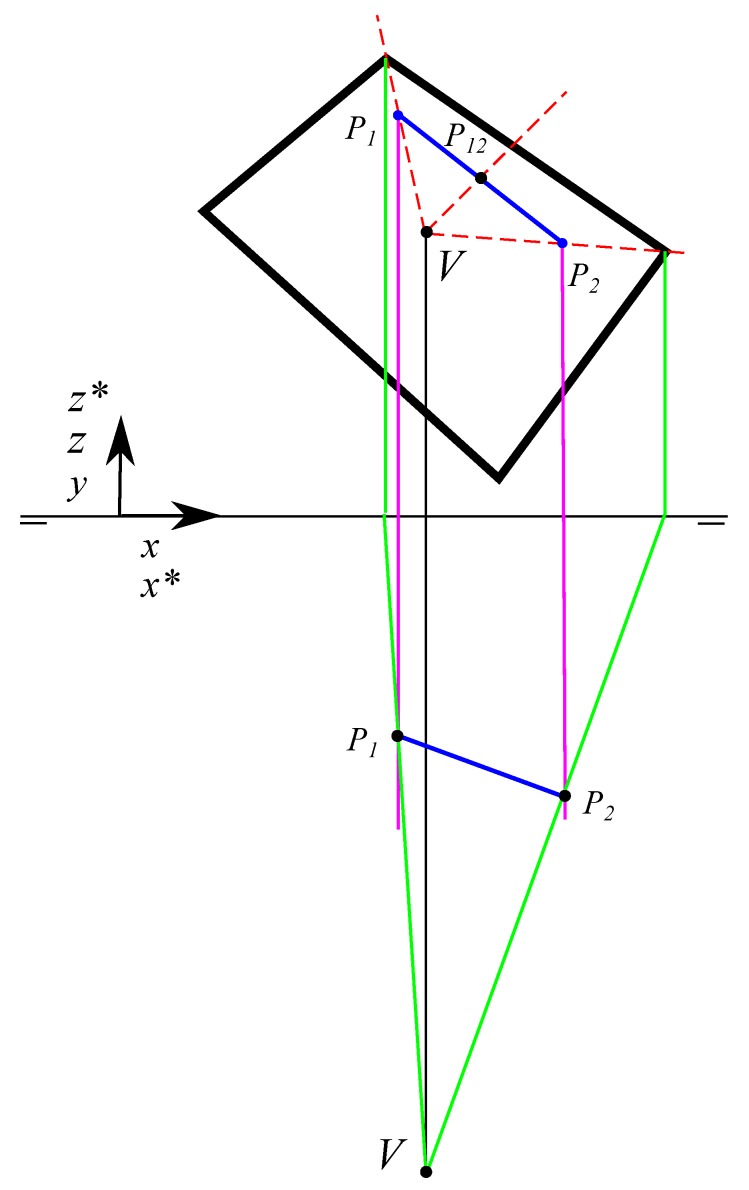
Obtaining the Top View from the Front View.

**Figure 6:**
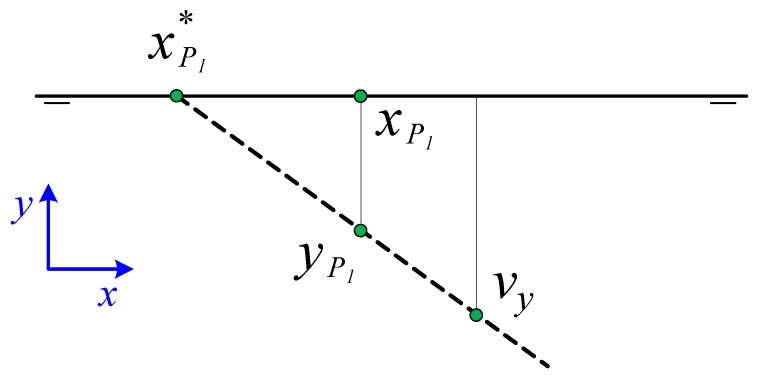
Obtaining the Top projection of a point of the conical with a known Front projection.

By taking advantage of these two properties and a trigonometric interrelation, a first proposal of a rectangle in Front View can be obtained by the following procedure:

1. In the triangle $P_{1}^{*}P_{2}^{*}V^{*}$ which is divided by the segment $V^{*}P_{12}^{*}$, a line that starts at $P_{12}$ and its intersection with the lines $V^{*}P_{1}^{*}$ and $V^{*}P_{2}^{*}$ is equidistant, in a way that a possible point $P_{12}$ in Front View can be obtained as shown in Figure 4.
2. An arbitrary distance *d* to obtain $P_{12}$ is taken.
3. The normal vector of the line $P_{1}P_{2}$ in dihedral will be found by a rotation of the vector $V^{*}P_{12}$ an angle ω reached using the trigonometric relation (1):
  (1) $$\tan\omega =\frac{2}{-\cot\alpha +\cot\beta .}$$
  The deduction of this expression is detailed in Appendix A, where α is the angle between $\vec{V^{*}P_{2}^{*}}$ and $\vec{V^{*}P_{12}^{*}}$; and β is the angle between $\vec{V^{*}P_{1}^{*}}$ and $\vec{V^{*}P_{12}^{*}}$ represented in the Figure 4 and expressed by the Equations (2) and (3).
  (2) $$\alpha =\arccos\frac{\vec{V^{*}P_{2}^{*}}\cdot \vec{V^{*}P_{12}^{*}}}{|\vec{V^{*}P_{2}^{*}}\left|\right|\vec{V^{*}P_{12}^{*}}|}$$
  (3) $$\beta =\arccos\frac{\vec{V^{*}P_{1}^{*}}\cdot \vec{V^{*}P_{12}^{*}}}{|\vec{V^{*}P_{1}^{*}}\left|\right|\vec{V^{*}P_{12}^{*}}|.}$$
4. Points $P_{1}$ and $P_{2}$ are obtained from the intersection of the line defined by the point $P_{12}$ and the vector $\vec{V^{*}P_{12}}$ rotated an angle ω. Once the orientations are calculated, starting from a point in the line $V^{*}P_{1}^{*}$ and drawing a line that intersects the line $V^{*}P_{2}^{*}$ gets the hypothetical side $P_{1}P_{2}$ already in the Front View of the dihedral projection.
5. With the presumed points $P_{1}$ and $P_{2}$ of the Front View in the dihedral projection, it is possible to calculate, with the central point *V* in the Top view (which is at a distance equal to the focal distance from the drawing plane), the projection in Top view of points $P_{1}$ and $P_{2}$ as shown in Figure 5.
  To get the Top View of the rectangle from the hypothetical Front projection of the side $P_{1}P_{2}$ it is possible to obtain its Top projection from the Top projection of *V* which is at a focal distance from the drawing plane. As an example, the *y* coordinate of the point $P_{1}$ will be obtained by the intersection of the line that joins *V* in the Top View with the point $x_{P1}^{*}$ and the coordinate $x_{P1}$ as shown in Figure 6 and in Equation (4). In the same way we proceed to obtain the *y* coordinate of the point $P_{2}$.
  (4) $$y_{p1}=V_{y}+\frac{V_{y}}{V_{x}-x_{P1}^{*}}$$
6. With the Top and Front projection of the points $P_{1}$ y $P_{2}$, the length in pixel units of the segment is calculated, and the real distance *d* that means the length $P_{1}P_{2}$ matched with the length of the edge of the rectangle is obtained.
  With the complete coordinates of the points $P_{1}$ y $P_{2}$ the distance between both points is calculated.
  Being a proportional geometric problem, the solution is found in a single step from the application of Thales’ theorem.
7. A similar procedure is done with the triangle $P_{2}^{*}P_{3}^{*}V^{*}$ divided by $V^{*}P_{23}$. Consequently, the two orientations of the edges which will have the projection of the rectangle in the dihedral system are calculated.
  Getting the rest of the points is direct as we have the orientations in Top View, finding the points $P_{3}$ y $P_{4}$ using a correlative method.

**Figure 7:**
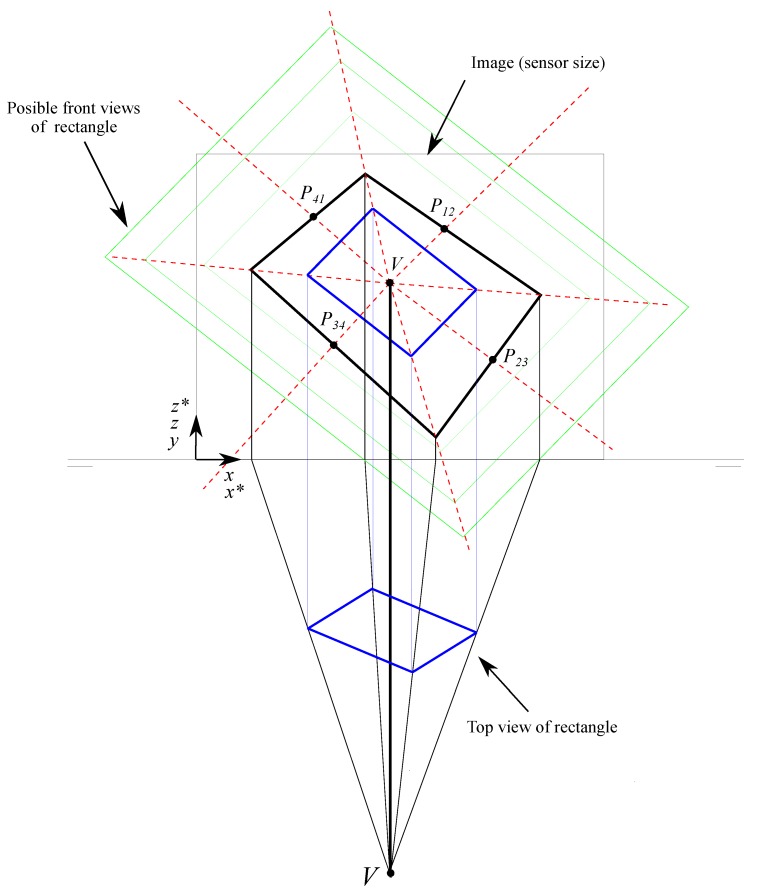
Summary for obtaining the dihedral from the conical projection (blue: dihedral; black: conical).

Once the three-dimensional coordinates of the rectangle are found, it is possible to perform any operation related to positioning and orientation of the camera or distance calculation and angle modification. The full method is represented in Figure 7.

## 5. Comments on the Described Method and Comparison with Previous Ones

The new method has many advantages over the methods of Haralik [8] and Wefelscheid [32] which are the most used:

- It has the advantage of working with points and lines as it works in descriptive geometry science, making the calculations much more intuitive, based on simple sequences.
- The method makes little use of trigonometric functions. The only trigonometric relation used is the tangent angle function between two vectors which induces very few floating point errors. In addition to this, a rotation is applied on the vectors to redraw the rectangle edges in dihedral.
- It is a direct method without iterations or matrix inversions.
- As it is sequential, we can perform checks and easily determine where an error may have occurred. Once the calculations have been verified, the equations in mega formulas that save the calculation times can be exposed. The algebraic operations to obtain the points barely exceed one hundred which equates to less than thousandths of a second of computer time.

With the results, several verification can be performed since it provides data which can already be calculated such as:

- The length of the second edge of the rectangle, since it has not been used for the calculation of the inverse perspective.
- The spatial lines that join the point *V* with the vanishing points $V_{1}$ and $V_{2}$ in the drawing plane, are parallel to the sides of the rectangle so they are perpendicular to each other. Consequently, the scalar product must be zero, which means that the starting data (the focal length) can actually be determined from the vanishing points [34].

## 6. Positioning of the Camera in Coordinate System Defined in the Rectangle

**Figure 8:**
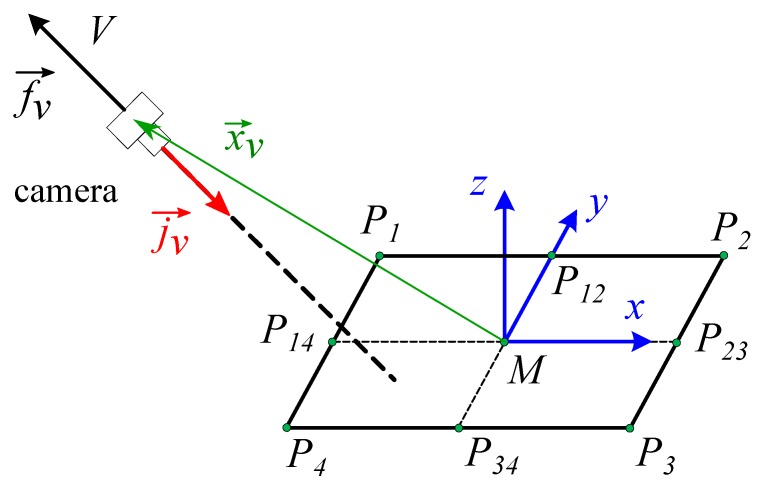
Obtaining camera positioning with respect to rectangle coordinate system.

Object tracking systems in space through images, navigation systems or calculation of distances and angles in images can be easily made from the coordinates of known rectangles that serve as a reference (Figure 8). Therefore, they can be used for tracker systems in positioning of parts in specific coordinate systems.

In global coordinates (5):

(5) $$\vec{i}=\frac{\vec{P_{1}P_{2}}}{|\vec{P_{1}P_{2}}|}\;\vec{j}=\frac{\vec{P_{4}P_{1}}}{|\vec{P_{4}P_{1}}|}\;\vec{k}=\frac{\vec{P_{1}P_{2}}\wedge \vec{P_{4}P_{1}}}{|\vec{P_{1}P_{2}}\wedge \vec{P_{4}P_{1}}|}=\vec{i}\wedge \vec{j}.$$

The position in global coordinates would be given by (6):

(6) $$\vec{X_{v}}=\left\{\begin{array}{c} x_{v}-x_{M}\\ y_{v}-y_{M}\\ z_{v}-z_{M} \end{array}\right\},$$

and the check in local coordinates would be obtained according to (7):

(7) $$\vec{X_{v}}=\left\{\begin{array}{c} \vec{MV}\cdot \vec{i}\\ \vec{MV}\cdot \vec{j}\\ \vec{MV}\cdot \vec{k} \end{array}\right\}.$$

In global coordinates the orientation of the camera follows the ${\vec{j}}_{v}$ vector (8):

(8) $$\left\{\begin{array}{c} 0\\ 1\\ 0 \end{array}\right\}{\vec{j}}_{v}.$$

In rectangle coordinates (9) is obtained, which is the projection of *j* on each of the three axes that coincides with the component in *y* of the three vectors $\vec{i}$, $\vec{j}$, and $\vec{k}$ expressed in global coordinates.

(9) $$\left\{\begin{array}{c} j_{vx}\\ j_{vy}\\ j_{vz} \end{array}\right\}=\left\{\begin{array}{c} \vec{i}\cdot {\vec{j}}_{v}\\ \vec{j}\cdot {\vec{j}}_{v}\\ \vec{k}\cdot {\vec{j}}_{v} \end{array}\right\}.$$

## 7. Experimental Tests

**Figure 9:**
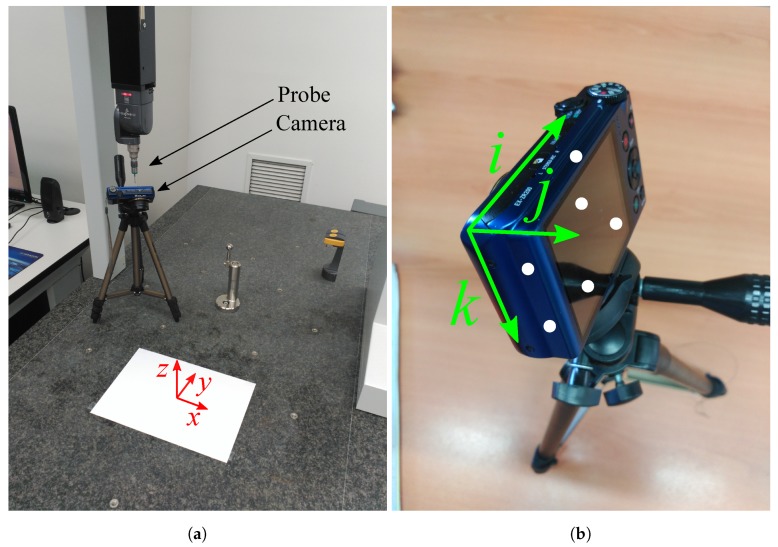
(**a**) Set-up used for the measurements of the position of the camera in rectangle coordinates through a CMM; (**b**) Points of the camera touched by the probe for the implementation of the 3-2-1 method [44].

For the test of the method all the images are taken with a CASIO Exilim EX-ZR200 digital camera with a resolution of 4608 × 3456 (16MPixels) and a sensor dimension of 6.16 × 4.62 mm (1/2.3”). After the calibration the focal distance is 4.6 mm and the central point is not in the middle of the image but at coordinates (2186, 1991). The camera is calibrated by standard method of Computer Vision by images taken of a chessboard. To analyze the position a Coordinate Measuring Machine (CMM) model Pioneer DEA 03.10.06 with measuring strokes 600 × 1000 × 600 mm has been used as seen in Figure 9. The Maximum Permissible Error of the DEA in the measurements is 2.8+4.0 L/1000 μm. The software for the measurements was PC-DMIS.

The procedure followed was as follows:

1. Place a DIN A4 size paper on the granite table.
2. Position the camera on a tripod.
3. Take photo of DIN A4 paper remotely so as not to influence the captured image.
4. Take six points of the camera housing according to the 3-2-1 method [44].
5. Calculate the position of the camera focus with respect to the center of the A4 sheet from the palpated points.
6. Contrast this position with that obtained by image analysis.
7. Repeat several times the steps 3–6, while varying the position and angles of the camera.

The parameters used for the test are summarized in Table 2. The coordinates and distances of the paper center obtained by CMM and the image analysis including the differences of the coordinates and distances between both, where subindex *e* refers to experimental data calculated with the CMM and subindex *t* refers to theoretical values calculated by the image analysis algorithm, are shown in the Table 3.

Result comments:

- The differences in measured distances are less than 2%, e.g., less than 2 cm in 1 m distance.
- The errors in the *x* coordinate are due to the parallelism of the lines with the image plane, but they hardly affect the distance calculation since the contribution of the *x* coordinate is small in the global calculation.

**Figure 10:**
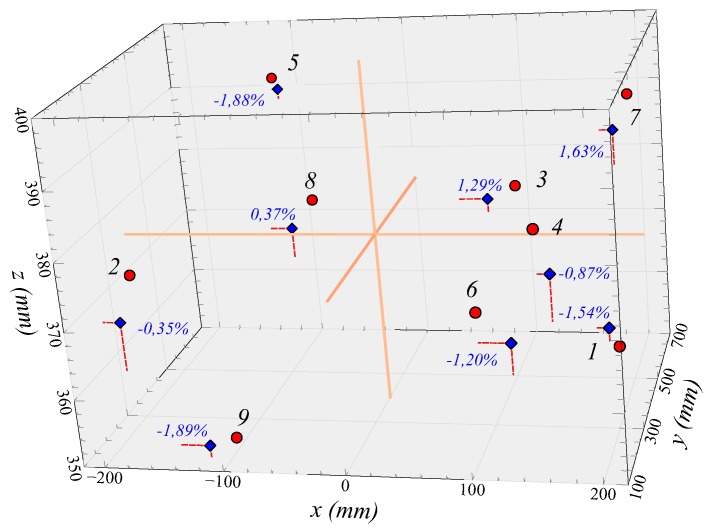
Graphical representation of the theoretical and experimental values of the center points of the rectangles. Distance errors have also been included for each point.

As can be seen in Table 3 and Figure 10, the difference between the distance from the camera to the center of the folio, measured by the image analysis and by the CMM, is less than 2%. This indicates that, visually, very close accuracies of the actual distances can be achieved. Nevertheless, analyzing each coordinate, a very high error in the *x* coordinates is observed in some points. These errors occur when the camera is facing the folio, being the x-axis parallel to the sensor plane. In consequence, the vanishing points in this direction are far apart, and the intersection between the lines is more imprecise. As in these cases, the camera is located at a small value of the x coordinate, the influence of this value on the global error is reduced. This indicates a limitation since the method works best, the closer the vanishing points remain.

**Figure 11:**
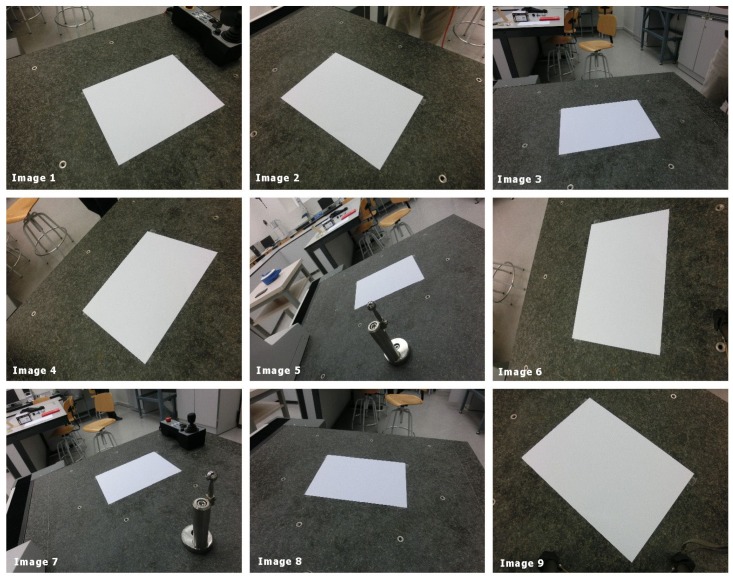
Images analyzed during the experimental test.

Figure 11 shows the images obtained with the camera and used to verify the method presented in this article.

## 8. Conclusions

A new method has been proposed for rectangle reconstruction using elements of descriptive geometry, as used by Monge in 1847 [42], and of extensive knowledge by engineering users since it is taught in the early stages of such studies. The method presented is mainly based on the intersection between lines, as their calculations are fast and stable in computing and, therefore, minimize errors and optimize computation. The proposed process uses very few trigonometric functions of small angles that are the main source of errors in other methods, so very few floating-point errors are introduced. Additionally, the trigonometric functions are mainly used for the rotation of vectors to align the edges in dihedral projections, which also reduces the errors.

In addition, a procedure was carried out to experimentally test the calculations. The proposed technique was tested in a CMM by locating the camera through the palpation using the 3-2-1 method and the position given by the CMM was compared with the calculation from the image taken by the camera. The proposed method provides maximum errors of 2% in the measured distances. The big errors detected in individual coordinates are due to the parallelism of two sides with the image plane since, in this case, the vanishing point is distance in space and its determination by the intersection of two almost parallel lines has more variability.

## Figures and Tables

**Table 1:** Obtaining auxiliary points as intersections of lines that go through two points.

Support Point	Line 1		Line 2	
$M^{*}$	$P_{1}^{*}$	$P_{3}^{*}$	$P_{2}^{*}$	$P_{4}^{*}$
$V_{1}^{*}$	$P_{1}^{*}$	$P_{2}^{*}$	$P_{3}^{*}$	$P_{4}^{*}$
$V_{2}^{*}$	$P_{1}^{*}$	$P_{4}^{*}$	$P_{2}^{*}$	$P_{3}^{*}$
$P_{12}^{*}$	$P_{1}^{*}$	$P_{2}^{*}$	$M^{*}$	$V_{2}^{*}$
$P_{23}^{*}$	$P_{2}^{*}$	$P_{3}^{*}$	$M^{*}$	$V_{1}^{*}$
$P_{34}^{*}$	$P_{3}^{*}$	$P_{4}^{*}$	$M^{*}$	$V_{2}^{*}$
$P_{14}^{*}$	$P_{1}^{*}$	$P_{4}^{*}$	$M^{*}$	$V_{1}^{*}$

**Table 2:** Parameters used for the test.

Camera Parameters			A4 Sheet Parameters			Focus Position in Camera Coordinates		
Sensor size	1/2.3”	6.16 × 4.62 mm	width	210	mm	x	30	mm
Width	4608	pixels	height	297	mm	y	30	mm
Height	3456	pixels				z	3.5	mm
Focus	3433	pix (4.6 mm)						
$V_{x}$	2186	pixels						
$V_{y}$	1991	pixels						
1 pixel	0.0013368	mm						

**Table 3:** Coordinates, distances, and errors obtained by CMM and image analysis where subindex *e* refers to experimental data calculated with the CMM and subindex *t* refers to theoretical values calculated by the image analysis algorithm. Coordinates *x*, *y*, *z* and distances *d* are expressed in mm. Differences of the coordinates and distances (errors) are presented in percentages.

	CMM				Image							
Image	$x_{e}$	$y_{e}$	$z_{e}$	$d_{e}$	$x_{t}$	$y_{t}$	$z_{t}$	$d_{t}$	$\epsilon_{x}$ (%)	$\epsilon_{y}$ (%)	$\epsilon_{z}$ (%)	$\epsilon_{d}$ (%)
1	201.22	222.21	367.44	474.21	210.09	200.44	365.64	466.91	4.41	−9.79	−0.49	−1.54
2	−195.04	218.71	367.43	469.98	−180.47	215.48	374.60	468.32	−7.47	−1.48	1.95	−0.35
3	88.39	469.44	378.18	609.27	111.54	473.32	380.00	617.15	26.19	0.83	0.48	1.29
4	157.90	225.84	374.79	465.19	148.48	212.45	381.40	461.13	−5.97	−5.93	1.76	−0.87
5	−113.73	672.02	390.02	785.27	−120.61	687.26	391.43	800.06	6.06	2.27	0.36	1.88
6	128.21	190.00	366.23	432.05	102.81	185.02	370.68	426.85	−19.82	−2.62	1.21	−1.20
7	184.55	592.80	384.91	730.50	197.23	600.11	389.99	742.38	6.88	1.23	1.32	1.63
8	−80.07	460.32	374.07	598.53	−60.11	462.76	378.33	600.75	−24.93	0.53	1.14	0.37
9	−113.76	128.64	352.65	392.24	−89.32	120.79	354.29	384.83	−21.48	−6.10	0.47	−1.89

## Abbreviations

The following abbreviations are used in this manuscript:

UAV	Unmanned Aerial Vehicle
CMM	Coordinate Measurement Machine
DIN	Deutsches Institut für Normung (German Institute for Standardization)

## Appendix A. Obtaining the Expression of tanω

In order to identify the sign of α and β the vector product is calculated and the corresponding sign is found (A1) and (A2):

(A1) $$\sin\alpha =\frac{\vec{V^{*}P_{2}^{*}}\wedge \vec{V^{*}P_{12}^{*}}}{|\vec{V^{*}P_{2}^{*}}\left|\right|\vec{V^{*}P_{12}^{*}}|}$$

(A2) $$\sin\beta =\frac{\vec{V^{*}P_{1}^{*}}\wedge \vec{V^{*}P_{12}^{*}}}{|\vec{V^{*}P_{1}^{*}}\left|\right|\vec{V^{*}P_{12}^{*}}|}.$$

The angle ω can be obtained from α and β applying the sine law (A3):

(A3) $$\frac{L}{2\sin\alpha }=\frac{|\vec{V^{*}P_{12}^{*}}|}{\sin(\pi -\alpha -\omega )}=\frac{|\vec{V^{*}P_{12}^{*}}|}{\sin\alpha \;\cos\omega +\cos\alpha \;\sin\omega }$$

(A4) $$\frac{L}{2\sin\beta }=\frac{|\vec{V^{*}P_{12}^{*}}|}{\sin(\omega -\beta )}=\frac{|\vec{V^{*}P_{12}^{*}}|}{\sin\omega \;\cos\beta +\cos\omega \;\sin\beta }$$

(A5) $$\frac{L\cos\omega }{2\sin\alpha }=\frac{|\vec{V^{*}P_{12}^{*}}|}{\sin\alpha +\cos\alpha \;\tan\omega }$$

(A6) $$\frac{L\cos\omega }{2\sin\beta }=\frac{|\vec{V^{*}P_{12}^{*}}|}{\cos\beta \;\tan\omega -\sin\beta }$$

(A7) $$\frac{L\cos\omega }{2}=\frac{|\vec{V^{*}P_{12}^{*}}|}{1+\cot\alpha \;\tan\omega }=\frac{|\vec{V^{*}P_{12}^{*}}|}{\cot\beta \;\tan\omega -1}$$

(A8) 1+cotαtanω=cotβtanω−1

(A9) 2+cotαtanω=cotβtanω.

Consequently, the angle of rotation ω can be calculated using the Equation (A10):

(A10) $$\tan\omega =\frac{2}{-\cot\alpha +\cot\beta }.$$

## References

[1] Duan F., Fuchao W., Hu Z. (2008). Pose determination and plane measurement using a trapezium. Pattern Recognit. Lett..

[2] Magee M., Aggarwal J. Determining the position of a robot using a single calibration object. Proceedings of the IEEE International Conference on Robotics and Automation.

[3] Gao X.H., Liang B., Pan L., Li Z.H., Zhang Y.C. (2016). A Monocular Structured Light Vision Method for Pose Determination of Large Non-cooperative Satellites. Int. J. Control. Autom. Syst..

[4] Martinez C., Mondragon I.F., Olivares-Mendez M.A., Campoy P. (2011). On-board and Ground Visual Pose Estimation Techniques for UAV Control. J. Intell. Robot. Syst..

[5] Zhang L., Zhai Z., He L., Wen P., Niu W. (2019). Infrared-Inertial Navigation for Commercial Aircraft Precision Landing in Low Visibility and GPS-Denied Environments. Sensors.

[6] Abidi M.A., Chandra T. (1995). A new efficient and direct solution for pose estimation using quadrangular targets—Algorithm and evaluation. IEEE Trans. Pattern Anal. Mach. Intell..

[7] Haralick R.M. (1980). Using perspective transformations in scene analysis. Comput. Graph. Image Process..

[8] Haralick R.M. (1989). Determining camera parameters from the perspective projection of a rectangle. Pattern Recognit..

[9] Quan L., Lan Z. (1999). Linear N-point camera pose determination. IEEE Trans. Pattern Anal. Mach. Intell..

[10] Sim R., Little J. (2009). Autonomous vision-based robotic exploration and mapping using hybrid maps and particle filters. Image Vis. Comput..

[11] Valencia-Garcia R., Martinez-Béjar R., Gasparetto A. (2005). An intelligent framework for simulating robot-assisted surgical operations. Expert Syst. Appl..

[12] Pichler A., Akkaladevi S., Ikeda M., Hofmann M., Plasch M., Wögerer C., Fritz G. (2017). Towards Shared Autonomy for Robotic Tasks in Manufacturing. Procedia Manuf..

[13] González D., Pérez J., Milanés V. (2017). Parametric-based path generation for automated vehicles at roundabouts. Expert Syst. Appl..

[14] Sanchez-Lopez J., Pestana J., De La Puente P., Campoy P. (2015). A reliable open-source system architecture for the fast designing and prototyping of autonomous multi-UAV systems: Simulation and experimentation. J. Intell. Robot. Syst..

[15] Romero-Ramirez F.J., Muñoz-Salinas R., Medina-Carnicer R. (2018). Speeded up detection of squared fiducial markers. Image Vis. Comput..

[16] Germanese D., Leone G.R., Moroni D., Pascali M.A., Tampucci M. (2018). Long-Term Monitoring of Crack Patterns in Historic Structures Using UAVs and Planar Markers: A Preliminary Study. J. Imaging.

[17] Pflugi S., Vasireddy R., Lerch T., Ecker T., Tannast M., Boemke N., Siebenrock K., Zheng G. Augmented marker tracking for peri-acetabular osteotomy surgery. Proceedings of the 2017 39th Annual International Conference of the IEEE Engineering in Medicine and Biology Society (EMBC).

[18] Lima J.P., Roberto R., Simões F., Almeida M., Figueiredo L., Teixeira J.M., Teichrieb V. (2017). Markerless tracking system for augmented reality in the automotive industry. Expert Syst. Appl..

[19] Chen P., Peng Z., Li D., Yang L. (2016). An improved augmented reality system based on AndAR. J. Vis. Commun. Image Represent..

[20] Khattak S., Cowan B., Chepurna I., Hogue A. A real-time reconstructed 3D environment augmented with virtual objects rendered with correct occlusion. Proceedings of the 2014 IEEE Games Media Entertainment Toronto.

[21] Shang Y., Yu Q., Zhang X. (2004). Analytical method for camera calibration from a single image with four coplanar control lines. Appl. Opt..

[22] Cai Y., Huang Y. (2013). A Robust Linear Camera Calibration Based on Coplanar Circles. Proceedings of 2013 Chinese Intelligent Automation Conference: Intelligent Information Processing. Chinese Assoc Automat, Intelligent Automat Comm.

[23] Takahashi A., Ishii I., Makino H., Nakashizuka M. (1996). A camera calibration method using parallelogrammatic grid points. IEICE Trans. Inf. Syst..

[24] Mozerov M., Amato A., Al Haj M., Gonzalez J. (2007). A Simple Method of Multiple Camera Calibration for the Joint Top View Projection. Computer Recognition Systems 2.

[25] Becker S., Bove V. Semiautomatic 3-D model extraction from uncalibrated 2-D camera views. Visual data exploration and analysis II. Proceedings of the Society of Photo-Optical Instrumentation Engineers (SPIE).

[26] Delage E., Lee H., Ng A.Y. (2007). Automatic single-image 3D reconstructions of indoor Manhattan world scenes. Robot. Res..

[27] Wilczkowiak M., Boyer E., Sturm P. (2001). Camera calibration and 3D reconstruction from single images using parallelepipeds. Proceedings of the 8th IEEE International Conference on Computer Vision.

[28] Sturm P., Maybank S. A Method for Interactive 3D Reconstruction of Piecewise Planar Objects from Single Images. Proceedings of the 10th British Machine Vision Conference (BMVC ’99).

[29] Micusik B., Wildenauer H., Kosecka J. (2008). Detection and matching of rectilinear structures. Proceedings of the 2008 IEEE Conference on Computer Vision and Pattern Recognition.

[30] Penna M. (1991). Determining camera parameters from the perspective projection of a quadrilateral. Pattern Recognit..

[31] Hong Z.Q., Yang J.Y. (1993). An algorithm for camera calibration using a three-dimensional reference point. Pattern Recognit..

[32] Wefelscheid C., Wekel T., Hellwich O. (2011). Monocular Rectangle Reconstruction Based on Direct Linear Transformation. Proceedings of the VISAPP 2011: Proceedings of the International Conference on Computer Vision Theory and Applications.

[33] Shunliang P., Xiaojian W., Weiqun S., Zishan S. (2006). A faster relative 3D position and attitude algorithm based on special four-point feature. Signal Analysis, Masurement Theory, Photo-Electronic Technology, and Artificial Intellingence, Pts 1 and 2.

[34] Guillou E., Meneveaux D., Maisel E., Bouatouch K. (2000). Using vanishing points for camera calibration and coarse 3D reconstruction from a single image. Vis. Comput..

[35] Zhou K., Wang X.J., Wang Z., Wei H., Yin L. (2018). Complete Initial Solutions for Iterative Pose Estimation From Planar Objects. IEEE Access.

[36] YUAN J. (1989). A General Photogrammetric Method for Determining Object Position and Orientation. IEEE Trans. Robot. Autom..

[37] Wang P., Xu G., Cheng Y., Yu Q. (2019). Camera pose estimation from lines: A fast, robust and general method. Mach. Vis. Appl..

[38] Ulrich M., Wiedemann C., Steger C. CAD-Based Recognition of 3D Objects in Monocular Images. Proceedings of the 2009 IEEE International Conference on Robotics and Automation ICRA.

[39] Sakcak B., Bascetta L., Ferretti G. Model based Detection and 3D Localization of Planar Objects for Industrial Setups. Proceedings of the 13th International Conference on Informatics in Control, Automation and Robotics (ICINCO).

[40] Han P., Zhao G. (2015). CAD-based 3D objects recognition in monocular images for mobile augmented reality. Comput. Graph. UK.

[41] He Z., Jiang Z., Zhao X., Zhang S., Wu C. (2019). Sparse Template-Based 6-D Pose Estimation of Metal Parts Using a Monocular Camera. IEEE Trans. Ind. Electron..

[42] Monge G. (1847). Géométrie Descriptive.

[43] Adler A.A. (1912). The Theory of Engineering Drawing.

[44] Estrems M., Sánchez H., Faura F. (2003). Influence of Fixtures on Dimensional Accuracy in Machining Processes. Int. J. Adv. Manuf. Technol..

